# A Portable Three-Layer Compton Camera for Wide-Energy-Range Gamma-ray Imaging: Design, Simulation and Preliminary Testing

**DOI:** 10.3390/s23218951

**Published:** 2023-11-03

**Authors:** Jipeng Zhang, Xiong Xiao, Ye Chen, Bin Zhang, Xinhua Ma, Xianyun Ai, Jinglun Li

**Affiliations:** State Key Laboratory of NBC Protection for Civilian, Beijing 102205, China; xzyz343@126.com (J.Z.); xiaox1999@126.com (X.X.); chenye10@tsinghua.org.cn (Y.C.); zhangbin@sklnbcpc.cn (B.Z.); maxinhua@sklnbcpc.cn (X.M.); aixianyun@sklnbcpc.cn (X.A.)

**Keywords:** Compton camera, wide energy range, Monte Carlo simulation, scintillation detector, image reconstruction

## Abstract

(1) Background: The imaging energy range of a typical Compton camera is limited due to the fact that scattered gamma photons are seldom fully absorbed when the incident energies are above 3 MeV. Further improving the upper energy limit of gamma-ray imaging has important application significance in the active interrogation of special nuclear materials and chemical warfare agents, as well as range verification of proton therapy. (2) Methods: To realize gamma-ray imaging in a wide energy range of 0.3~7 MeV, a principle prototype, named a portable three-layer Compton camera, is developed using the scintillation detector that consists of an silicon photomultiplier array coupled with a Gd_3_Al_2_Ga_3_O_12_:Ce pixelated scintillator array. Implemented in a list-mode maximum likelihood expectation maximization algorithm, a far-field energy-domain imaging method based on the two interaction events is applied to estimate the initial energy and spatial distribution of gamma-ray sources. The simulation model of the detectors is established based on the Monte Carlo simulation toolkit Geant4. The reconstructed images of a ^133^Ba, a ^137^Cs and a ^60^Co point-like sources have been successfully obtained with our prototype in laboratory tests and compared with simulation studies. (3) Results: The proportion of effective imaging events accounts for about 2%, which allows our prototype to realize the reconstruction of the distribution of a 0.05 μSv/h ^137^Cs source in 10 s. The angular resolution for resolving two ^137^Cs point-like sources is 15°. Additional simulated imaging of the 6.13 MeV gamma-rays from 14.1 MeV neutron scattering with water preliminarily demonstrates the imaging capability for high incident energy. (4) Conclusions: We conclude that the prototype has a good imaging performance in a wide energy range (0.3~7 MeV), which shows potential in several MeV gamma-ray imaging applications.

## 1. Introduction

The Compton camera [1], which reconstructs images to visually display the spatial distribution of radioactive materials, has found extensive applications in astronomical observation [2], nuclear medicine [3], proton therapy [4,5], neutron activation imaging [6,7], and environmental measurement [8,9]. These applications benefit from the Compton camera’s advantages of a wider field of view (2π or 4π) and a wider energy range (from several hundreds of keV to a few MeV). In a Compton camera, the single scattering events occurring at two pixels of the detector are the main effective imaging events; that is, the incident gamma photon scatters at the first interaction position, and the scattered photon deposits all of its energy at the second interaction position. However, with the increase in energy, the probability of a scattered gamma photon being fully absorbed will decrease, and the probability of continuing scattering or escaping the detector will increase. In addition, the pair-production events that occur in high-energy gamma-ray imaging will cause the image artifacts to contaminate the image [10]. Therefore, the imaging energy range of a typical Compton camera usually ranges from 0.3 to 3 MeV.

Further improving the upper energy limit of traditional gamma-ray imaging has important application significance in many fields. In the active interrogation of special nuclear materials (SNM), the energy range of interest is between 2.5 and 6 MeV [11]. In the detection of chemical warfare (CW) agents using fast 14 MeV neutrons, measuring the ratios of chemical elements abundances, namely C/Cl, C/N, C/S, C/F, C/P, C/O, requires collecting gamma-rays within 8 MeV [12]. In the range verification in proton therapy, prompt gammas (PGs) are emitted from nuclear de-excitation in a characteristic spectrum, with a broad continuum ranging from 1 to 7 MeV [13], the most frequently used among them being 4.44 and 6.13 MeV gamma-rays, from the de-excitation of ^12^C* and ^16^O*, respectively.

To realize Compton imaging above 3 MeV, some improved methods on the basis of a scattering–absorption structure are adopted, such as adding scatterer layers and increasing the absorber volume [3,4,5,14]. A different approach is to consider more than two interaction events based on three parallel detector layers, which is called a three-Compton telescope [6,15]. In this method, incident gamma photons are scattered at two interaction positions in the first two layers of the detectors, and the second scattered photons do not need to be fully absorbed at the third interaction position in the third detector. In recent years, the IRIS group of IFIC-Valencia has developed and improved the prototype using this method for proton therapy treatment monitoring, which is called MACACO (Medical Applications CompAct COmpton camera) [16,17,18]. The prototype is composed of three detector layers, each made of a LaBr_3_ monolithic scintillator crystal coupled to a silicon photomultiplier (SiPM) array.

This article aims to realize gamma-ray imaging in a wide energy range of 0.3~7 MeV using three position-sensitive detector planes (Det1, Det2, and Det3). Considering the small size and easy handling of SiPM, as well as the advantages of the low cost and high detection efficiency of the scintillator, each detector plane is composed of a pixelated scintillator array coupled to a SiPM array. Ce-doped gadolinium–aluminum–gallium–garnet (Gd_3_Al_2_Ga_3_O_12_:Ce, GAGG:Ce) is selected as the scintillator material due to its characteristics of a high light yield and good energy resolution, being non-hygroscopic, and having no natural radioactivity [19]. The position-sensitive detector of a GAGG:Ce array coupled to a SiPM array has been widely applied in Compton imaging systems and has achieved good imaging results [3,4,7,8,9,20,21]. In our previous work, two types of GAGG-SiPM detector modules with varying thicknesses were designed to achieve three-Compton imaging in the energy range of 0.3~7 MeV [22]. Experimental testing and evaluation have been conducted on the position resolution and pixel energy resolution of the detector modules.

In this paper, the distance between three detector modules is further analyzed based on the Geant4 Monte Carlo simulation toolkit. To obtain the energy and direction of gamma-ray sources with a wide energy domain, spatial and spectral reconstruction is carried out for the two interaction events (2-events) according to the information of the interaction position and deposited energy. The principal prototype, named a portable three-layer Compton camera (TLCC), is developed on the basis of a detector module, data acquisition system, and image reconstruction algorithm. The imaging performance of the prototype is evaluated by using single point-like sources and multiple point-like sources under simulation and experimental conditions. Based on the emitted gamma-ray data coming from 14.1 MeV neutron scattering with water, the imaging ability of the system for a 6.13 MeV source is demonstrated via simulation. This work will provide a reference for research on Compton imaging technology under several MeV energies.

## 2. Materials and Methods

### 2.1. Wide-Energy-Range Gamma-ray Imaging

**Figure 1:**
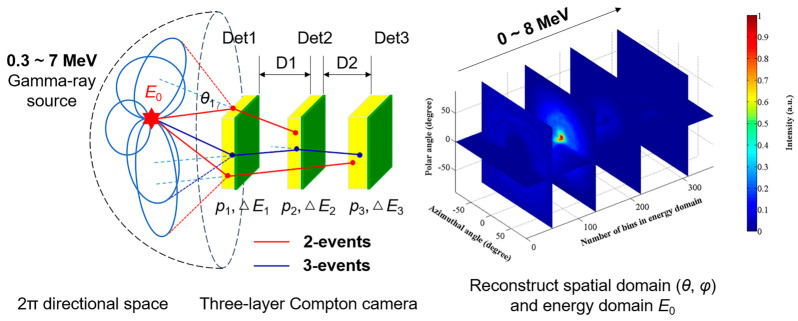
The principle diagram of far-field energy-domain imaging method for wide-energy-range gamma-ray imaging.

To reconstruct the incident direction of 0.3~7 MeV gamma-rays, the far-field energy-domain imaging method based on 2-events of TLCC is proposed, as shown in Figure 1. A 2-event is when a gamma-ray only interacts with the pixels of two detectors (Det1–Det2, Det1–Det3, or Det2–Det3). There are also three interaction events (3-events) under the three-layer detector structure (Det1–Det2–Det3), which are not used in our system because of their low probability of occurrence and the poor statistics of effective events. The sequence of 2-events is determined according to the physical order of the detectors. It is noteworthy that when using the 2-events to reconstruct images, the deposited energy in the scattering detector (*E*_s_) should be less than the backscattered photon energy at *θ* = 180° (*E*_bs_), that is, *E*_s_ < *E*_bs_ *= E* / (1 + 2*E* / *m*_e_*c*^2^), where *E* is the initial energy of the incident gamma-ray [22]. For different incident energies, the upper limit value of *E*_bs_ is 256 keV [22]. This is chosen because the influence of backscattering events should be eliminated as much as possible.

In Compton imaging, each event will create a cone with an opening apex angle derived from the Compton scattering formula, known as a Compton cone. The cone’s axis is given by the vector connecting the first two interaction positions. The surface of a Compton cone represents the possible original directions of the incident gamma photon. If the incident energy *E*_0_ and the energy loss at the first interaction Δ*E*_1_ are known, then the opening angle (the Compton scattering angle at the first interaction, *θ*_1_) is represented by:

(1) $$\cos\theta_{1}=1-m_{e}c^{2}(\frac{1}{E_{0}-\Delta E_{1}}-\frac{1}{E_{0}}),$$

where *m*_e_*c*^2^ is the rest mass of the electron.

Actually, there is no priori knowledge of the incident gamma-ray energy in some applications. Therefore, how to estimate the initial energy becomes the key to realize Compton imaging. For a 3-event, in which a gamma photon undergoes two successive Compton scatter interactions followed by a third interaction, the energy of the incident gamma photon can be calculated. If the interaction positions at the three interaction sites are *p*_1_, *p*_2_, and *p*_3_, the energy losses at each detector layer are Δ*E*_1_, Δ*E*_2_, and Δ*E*_3_, and the scattering angle at the first two interaction sites are *θ*_1_, *θ*_2_; then, the incident energy *E*_0_ can be calculated as:

(2) $$E_{0}=\Delta E_{1}+\frac{1}{2}\left(\Delta E_{2}+\sqrt{\Delta E_{2}{}^{2}+\frac{4m_{e}c^{2}\Delta E_{2}}{1-\cos\theta_{2}}}\right),$$

(3) $$\cos\theta_{2}=\frac{\vec{p_{1}p_{2}}\cdot \vec{p_{2}p_{3}}}{\left|\vec{p_{1}p_{2}}\right|\left|\vec{p_{2}p_{3}}\right|}.$$

However, the efficiency of collecting 3-events is significantly lower. It is necessary to estimate the possible incident energy according to the summation of the observed 2-events. In this paper, the spectral dimension is added to the emission distribution, and it is extended over both the spatial domain and energy domain. Thus, each event can obtain the emission energy from any value in the spectral range. Considering that the gamma-ray emission follows a Poisson distribution, the list-mode maximum likelihood expectation maximization (LM-MLEM) [23] is appropriate for reconstructing the initial energy and spatial distribution of gamma-ray sources through the iterative calculation. Here, the system matrix *t_ij_*_,*E*0_, presents the probability of an emission from the *j*th pixel under incident energy *E*_0_ to be detected as the *i*th event. The LM-MLEM algorithm is described as follows:

(4) $$\lambda_{j,E_{0}}{}^{n}=\frac{\lambda_{j,E_{0}}{}^{n-1}}{s_{j,E_{0}}}\sum_{i}\frac{t_{ij,E_{0}}}{\sum_{k}t_{ik,E_{0}}\lambda_{k,E_{0}}{}^{n-1}},$$

where λ*_j_*_,*E*0_*^n^* denotes the image value of pixel *j* after *n* iterations, *s_j_*_,*E*0_ is the sensitivity of the detector for pixel *j*. In this paper, the Compton cone of each event is expressed in a spherical coordinate system, and the imaging space for Equation (4) is the 2π directional space. Therefore, *s_j_*_,*E*0_ is assumed to be uniform in this work. The reconstructed result of gamma-ray direction is expressed by polar angle and azimuthal angle (*θ*, *φ*). When the source-to-detector distance is large compared to the detector size, the vertex of each Compton cone is approximately considered to be at the coordinate origin. Under the far-field approximation, the system matrix *t_ij_*_,*E*0_ is formulated as:

(5) $$t_{ij,E_{0}}=\frac{1}{\omega (\theta_{i,E_{0}})}\times \frac{1}{\sqrt{2\pi }\sigma }\exp\left[-\frac{{\left(\alpha_{ij,E_{0}}-\theta_{i,E_{0}}\right)}^{2}}{2\sigma^{2}}\right]\times p_{i}(E_{0}),$$

where *θ_i_*_,*E*0_ is the scattering angle calculated from Equation (1), *α_ij_*_,*E*0_ is the angle between the Compton cone axis and the direction of the interested image pixel *j* on sphere space, and *σ* is the Gaussian width of the cone (a constant small value correspond to the uncertainty of the cone angle). The first term of Equation (5) represents the weight factor of each event, in which the denominator *ω*(*θ_i_*_,*E*0_) is the integral of the Gaussian error function (erf(*θ_i_*_,*E*0_*/√2σ*)) over a bounded interval [0, π]; the second term represents a Gaussian distribution from the uncertainty of the cone; and the last term is the interaction probability of a photon with incident energy *E*_0_, which is divided into photopeak event and Compton-continuum event according to the second interaction. In Equation (5), *ω*(*θ_i_*_,*E*0_) and *p_i_*(*E*_0_) are described in detail as:

(6) $$\omega (\theta_{i,E_{0}})=\pi \left(\theta_{i,E_{0}}+\frac{\sqrt{\pi }\sigma }{2}\exp\left[-\frac{\theta_{i,E_{0}}{}^{2}}{2\sigma^{2}}\right]+\theta_{i,E_{0}}\times \mathrm{erf}\left(\frac{\theta_{i,E_{0}}}{\sqrt{2}\sigma }\right)\right),$$

(7) $$p_{i}(E_{0})=\left\{\begin{array}{l} \frac{1}{\sqrt{2\pi \sigma_{E_{1,i}}^{2}+\sigma_{E_{2,i}}^{2}}}\exp\left[-\frac{{\left(E_{0}-E_{1,i}-E_{2,i}\right)}^{2}}{2\left(\sigma_{E_{1,i}}^{2}+\sigma_{E_{2,i}}^{2}\right)}\right],\mathrm{if}\;E_{0}\in \left[E_{1,i}+E_{2,i}\pm 3\left(\sigma_{E_{1,i}}+\sigma_{E_{2,i}}\right)\right]\\ {\left.\frac{2\pi m_{e}c^{2}}{{\left(E_{0}-E_{1,i}-E_{2,i}\right)}^{2}}\frac{d\sigma_{C}\left(E_{0}-E_{1,i}\right)}{d\Omega }\right|}_{E_{2,i}},\mathrm{if}\;E_{0}>E_{1,i}+E_{2,i}+3\left(\sigma_{E_{1,i}}+\sigma_{E_{2,i}}\right) \end{array}\right.,$$

where *E*_1,*i*_, *E*_2,*i*_, σ*_E_*_1,*i*_, and σ*_E_*_2,*i*_ are the deposited energy and Gaussian standard deviation [24] of the first two interactions, respectively. In Equation (7), if the scattered gamma photon from the first interaction is fully absorbed at the second interaction, a Gaussian function is used to describe the situation (*E*_0_ ∈ [(*E*_1_ + *E*_2_) ± 3(σ*_E_*_1_ + σ*_E_*_2_)]). If the second interaction is a Compton scattering and the scattered photon escapes the detector, only part of the energy of the incident gamma-ray is deposited (*E*_0_ > (*E*_1_ + *E*_2_) + 3(σ*_E_*_1_ + σ*_E_*_2_)). The term dσ_C_(*E*_0_−*E*_1,*i*_)/dΩ|*_E_*_2,*i*_ presents the probability that the gamma photon with energy (*E*_0_−*E*_1_) deposits *E*_2_ in the second interaction via Compton scattering, which is predicted by the Klein–Nishina formula.

In the far-field energy domain imaging method, the spatial reconstruction part is similar to that proposed by Kishimoto A [3] and Omata A [21], and the energy domain part is similar to that proposed by Xu D [25] and Muñoz E [26]. In order to reduce the burden of matrix calculation in iterative reconstruction, the spatial and spectral imaging space are simplified appropriately. The number of bins in (*θ*, *φ*) spatial domain is set to 60 × 60, which means the angle accuracy of each image pixel is 3°. The number of bins in *E*_0_ energy domain is set to 360, which is an inter-partition distribution covering the energy range of 0 to 8 MeV, as shown in Equation (8).

(8) $$E_{0}{}_{bin}\left(i\right)=\left\{\begin{array}{l} \left(0.5+i\right)\times 5{,}_{}\mathrm{if}\;i<200\\ 1000+\left(0.5+\left(i-200\right)\right)\times 10{,}_{}\mathrm{if}\;200\leq i<300\\ 2000+\left(0.5+\left(i-300\right)\right)\times 100{,}_{}\mathrm{if}\;i>300 \end{array}\right..$$

### 2.2. Materials and Monte Carlo Simulation

**Figure 2:**
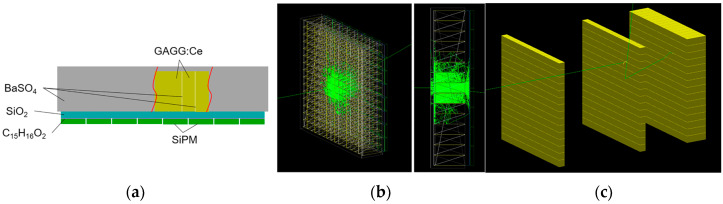
The detector simulation model constructed in Geant4. (**a**) The schematic of materials and placement. (**b**) The schematic of optical photon transportation in a single detector module. (**c**) The schematic of the three-layer detectors.

In the prototype of the TLCC, each layer is a position-sensitive detector composed of a GAGG:Ce scintillator array and a SiPM array. In order to improve the position resolution accuracy of the detector while taking into account the complexity of readout electronics, the number of pixels in the scintillator array is usually larger than in the SiPM array. An optical light guide with a certain thickness should be placed between the scintillator array and the SiPM array, so that the scintillation light generated by the interacting crystal pixel can be shared by neighboring SiPM pixels to avoid saturation of a single SiPM pixel. Based on the Geant4 Monte Carlo simulation toolkit [27], a detector simulation model including optical parameters of materials was constructed in our previous work [22], as shown in Figure 2.

In the simulation, the number of pixels in the SiPM array is set as 8 × 8 = 64, in which the effective area of a single pixel is 6 × 6 mm^2^ and the pixel gap is 0.2 mm. As such, the area of the SiPM array is 49.4 × 49.4 mm^2^. The thickness of the BaSO_4_ reflective layer between the pixels of the scintillator array is fixed at 0.1 mm. The spatial resolution and interaction event types of the detector are studied by changing the geometric parameters such as GAGG pixel size, pixel thickness, and light guide thickness. The physical process is defined using the PhysicsList class of Geant4, in which G4DecayPhysics class and BiasedRDPhysics class are selected to contain the radioactive decay process, G4EmStandardPhysics class is selected to contain the electromagnetic interaction process, and G4OpticalPhysics class is selected to contain the optical process. According to the above simulation study, the number and size of the pixels in GAGG are designed to be 15 × 15 and 3.2 × 3.2 mm^2^, respectively [22]. The pixel thickness is designed to be 3 mm or 10 mm, which corresponds to the first two detectors and the third detector, respectively [22]. The thickness of the light guide is expected to be 1.25 mm [22].

**Figure 3:**
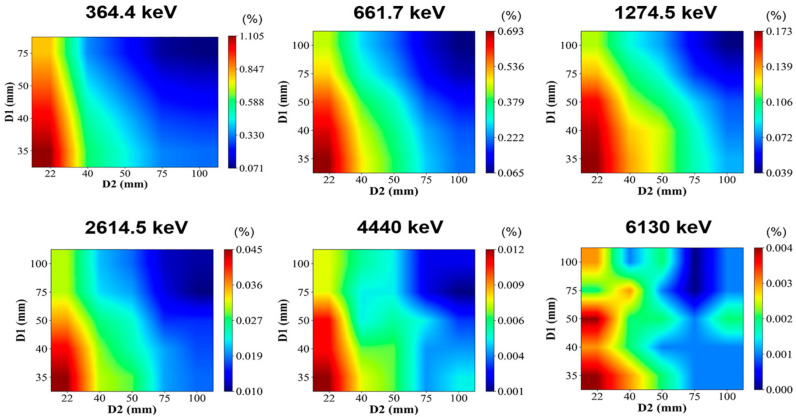
Percentage of effective 2-events of full absorption under different incident energies, given by using mono-energetic gamma-ray sources in Geant4.

In this work, the distance between the three-layer detectors (D1, D2) is further studied to maximize effective detection efficiency for a wide range of gamma-ray energies. In Geant4, the initial gamma-ray is generated using PrimaryGeneratorAction class. The point-like source with mono-energy of 364.4, 661.7, 1274.5, 2614.5, 4440, and 6130 keV is defined for ^131^I, ^137^Cs, ^22^Na, ^232^Th, and two high-energy gamma-ray emitters as an example, respectively, with a 1 m distance away from the center of the detector. In particular, the mono-energetic gamma-ray sources were used here to obtain the percentage of effective imaging events under different incident energies, which is calculated by the ratio of the counts of effective 2-events of full absorption to the counts of incident mono-energetic gamma-rays detected by the system. The results are shown in Figure 3. The effective 2-events of full absorption are defined as events in which the incident energy is completely deposited after the backscattering is excluded. It can be seen that the probability of full absorption decreases with the increase in D1 and D2, and the distance between the second and third layers (D2) has a more significant influence. Therefore, in order to maximize detection efficiency, and considering the detector module’s physical volume limitation, D2 is set to 22 mm.

**Figure 4:**
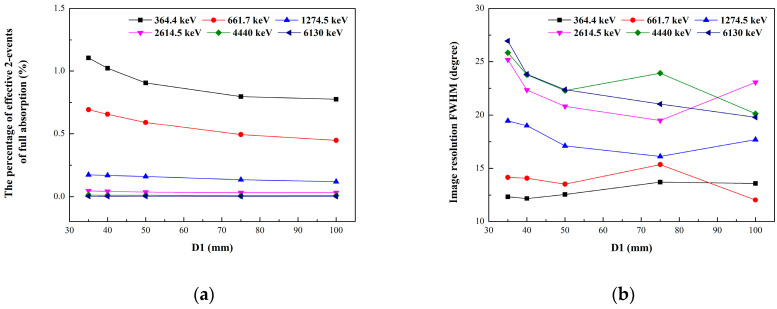
The curves of detection efficiency and angular resolution with the distance between the first and second layers (D1) at different incident energies when D2 = 22 mm. (**a**) The detection efficiency is indicated by the percentage of effective 2-events of full absorption. (**b**) The angular resolution is indicated by the full width half maximum (FWHM) of the reconstructed images.

Figure 4 shows the curves of the detection efficiency and angular resolution with the distance between the first and second layers (D1) at different incident energies when D2 = 22 mm. The detection efficiency is indicated by the percentage of effective 2-events of full absorption, and the angular resolution is indicated by the full width half maximum (FWHM) of the reconstructed images. In general, with the increase in D1, the detection efficiency will decrease, and the angular resolution will increase. However, due to the influence of event statistics and the imaging algorithm, the change in angular resolution is not as obvious as that in the detection efficiency. In this occasion, we set D1 = 40 mm to achieve a good balance between the efficiency and angular resolution of the system.

Based on the detector simulation model, the imaging performance of the prototype, such as detection efficiency, field-of-view (FOV), and angular resolution, can be evaluated and compared with experimental results. To match the real radionuclide testing conditions, the gamma photon will be emitted according to the probabilities per decay, i.e., for a ^60^Co nuclide, the energy of 1173.2 keV is emitted with 0.9985 probability, and the energy of 1332.5 keV is emitted with 0.999826 probability. The total emission gamma photon number *N* of a nuclide is obtained by multiplying the emission probability of each energy branch by the number 10^7^. The source-to-detector distance is set to 1 m. The emission direction of gamma-ray source is defined within a fixed solid angle instead of 4π to improve the calculation efficiency. In this work, the solid angle Ω is set to the angle corresponding to a circle with a radius of 10 cm at 1 m, which is enough to cover the area of the detector. In this occasion, Ω is calculated to be 0.031 sr.

### 2.3. The Principle Prototype and Data Acquisition

**Figure 5:**
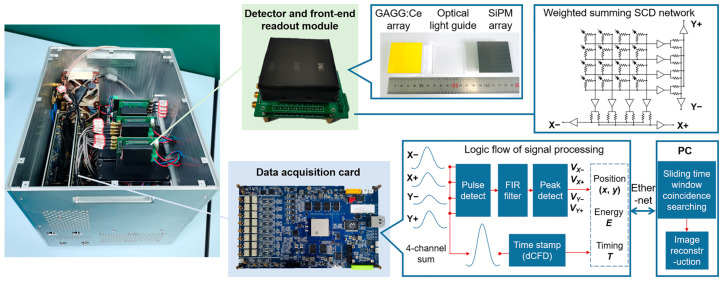
Photograph and signal processing flow of the three-layer Compton camera prototype.

The TLCC prototype, shown in Figure 5, is mainly composed of detector and front-end readout modules, the data acquisition system, and image reconstruction software. The system has three position-sensitive detectors: two scatterers and an absorber of GAGG:Ce scintillator (Epic Crystal Co., Ltd, Kunshan, JS 215332 China) with a density of 6.63 g/cm^3^. All the GAGG:Ce array blocks consist of 15 × 15 pixels separated by a 0.1 mm BaSO_4_ spacer. The GAGG:Ce array is coupled to a 8 × 8 SiPM array ArrayJ-60035-64P (Semiconductor Components Industries, LLC, Scottsdale, AZ 85250 USA), in which the sensitive area of a pixel is 6.07 × 6.07 mm^2^. The size of each GAGG:Ce element in the array is 3.2 × 3.2 × 3 mm^3^ for the scatterer and 3.2 × 3.2 × 10 mm^3^ for the absorber, respectively. There is a 1.25 mm thick SiO_2_ light guide coupled between the GAGG:Ce array and SiPM array. The periphery of the detector module is wrapped with Teflon and treated by light-shielding.

The weighted summing symmetric charge division (SCD) network [28] is used as the front-end readout circuit connected to the SiPM array, which achieves transferring 64-channel signals of the 8 × 8 array SiPM to 4-channel quasi-Gaussian signals. A total of 12 output signals from three detector modules are fed to the data acquisition card. After digital sampling by analog-to-digital converters (ADCs), the pulse detection, FIR filtering, and peak detection are completed in the field-programmable gate array (FPGA) to obtain the amplitude of the signal. The gamma-ray interaction position (*x*, *y*) and deposited energy *E* in a detector can be calculated by the amplitude of 4-channel signals [22]. The analog pulses are sampled at 25 ns intervals (40 MHz).

To acquire coincidence events of the system, it is necessary to obtain the time stamp of the signal by using the method of digital constant fraction discriminator (dCFD) in FPGA. In this method, the original 4-channel sum signal will be divided into two signals. One is delayed and another is inverted and attenuated by a factor. The sum of these two signals is a bipolar pulse with a zero-crossing point. The time stamp at zero-crossing is estimated by linear interpolation. The advantage of this method is that the trigger is independent on the signal peak height. Finally, the packaged data (position, energy, and timing) of interaction events are transmitted to a personal computer (PC) for coincidence event selection and image reconstruction via Ethernet. The sliding time window coincidence searching method is used to select coincidence events. The received event information is stored in three queues in a list mode according to the serial numbers of the three detectors. When the search starts, the time window is set to 2 clock ticks (50 ns), and each queue dequeues one event. Taking the minimum time stamp of three dequeued events as the starting time, it is judged whether the time stamps of the other two events are within the time window; if so, it is recorded as a coincidence event, and otherwise recorded as a single interaction event. Then, the next event in the queue continues to dequeue, and the above process is repeated. This method can select 2-events occurring on the three detectors in real time. The sequence of each selected event is rearranged according to the physical order of detectors.

**Figure 6:**
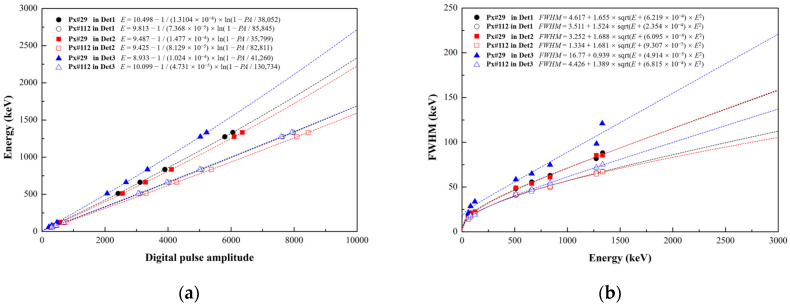
The spectral performance of pixel No.29 and pixel No.112 in the three detectors at eight energies (59.5 keV, 81 keV, 122 keV, 511 keV, 661.7 keV, 834.8 keV, 1274.5 keV, 1332.5 keV). (**a**) The photopeak energies (keV) as functions of digital pulse amplitude. (**b**) The measured *FWHM*s of the photopeak energies and fitting curves.

In order to realize image reconstruction, the detector characterization should be carried out first to obtain the calibration value of deposited energy on each pixel. All the measurements are conducted inside an air-conditioned room. The bias voltage of SiPM is fixed to 28.5 V, and the temperature maintains a 22 °C constant temperature to avoid variations in the SiPM’s response. Considering that the maximum number of photons that can be received by the photosensitive elements of a SiPM per unit time is limited, the linearity of the detector will become worse in the high-energy region. Therefore, the logarithmic function is used to fit the energy and SiPM’s pulse amplitude. The fitting function is:

(9) $$E=a-\frac{1}{b}\ln\left(1-\frac{PA}{c}\right),$$

where *PA* is the digital pulse amplitude of SiPM and *E* is the energy with keV units. Taking pixel No. 29 and pixel No. 112 as the representatives of the edge region and the central region, respectively, the relationships between the pulse amplitudes and photopeak energies of 59.5 keV, 81 keV, 122 keV, 511 keV, 661.7 keV, 834.8 keV, 1274.5 keV, and 1332.5 keV in the three detectors are shown in Figure 6a. After the energy calibration of pixels in the three detectors is completed, the energy resolution of pixels is calculated as the ratio of the FWHM to the photopeak energies. The FWHM is defined as 2.35σ of the Gaussian distribution obtained by Gaussian fitting. As shown in Figure 6b, the measured FWHMs of the photopeak energies can be fitted as follows:

(10) $$FWHM=a+b\sqrt{E+cE^{2}}$$

The averaged energy resolution of the pixels is measured as (7.47 ± 0.58)%, (7.43 ± 0.61)%, and (8.37 ± 0.87)% at 661.7 keV for the three detectors, respectively. The data of pixel energy resolution in the three detectors is also used in G4UserEventAction class of the Geant4 simulation model to reproduce the experimental behavior of the prototype.

## 3. Results

### 3.1. Single-Source Imaging and Performance Evaluation

**Figure 7:**
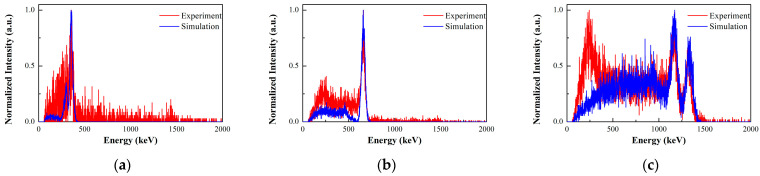
Comparison between the experimental measurements and simulated data for the coincidence summed energy spectra of the 2-events: (**a**) ^133^Ba source; (**b**) ^137^Cs source; (**c**) ^60^Co source.

In this paragraph, we present the results of experimental measurements and simulated data for several single point-like sources (^133^Ba, ^137^Cs, ^60^Co) to validate the imaging performance of the prototype, such as detection efficiency, field of view, and imaging sensitivity. The activities of the point-like sources are 0.262 MBq, 1.88 MBq, and 1.92 MBq, respectively. The coincidence summed energy spectra of the 2-events in the prototype are shown in Figure 7, in which both the experimental and simulation results are displayed. For the experimental spectra of coincidence events, the natural background spectra in the same acquisition time are deducted. The vertical axis in the figure is represented by the normalized intensity. For all the radionuclides, the photopeaks can be clearly observed, and the intensity distribution of photopeaks is in good agreement with the simulation. In the low-energy range of less than 500 keV, more coincidence events are obtained in the experiment than in the simulation. We expect that this is caused by the influence of the scattering from the external environment around the detector.

#### 3.1.1. Detection Efficiency and Image Resolution

The detection efficiency in the Compton imaging system is defined as the proportion of effective imaging events in the measured events for the incident gamma-rays. In the experimental measurements, the measured counts of incident gamma-rays are equal to the total counts recorded by the system minus the counts of natural background, which means that the single interaction, two interactions, and three interactions of the initial gamma-rays are included. As described in this paper, the count rate of the natural background is 198 cps (57.5 cps, 49 cps, and 91.5 cps for Det1, Det2, and Det3, respectively).

Table 1 summarizes the detection efficiency evaluation results from three point-like sources (^133^Ba, ^137^Cs, ^60^Co) positioned at the center of the FOV under the conditions of simulation and experiment. For the simulation, the gamma-ray emitter with a solid angle Ω = 0.031 sr is placed 1 m away from the front of the detector. The emission number is given by the sum of the emission probability of each energy branch multiplied by the number 10^7^. For the experiments, the detailed testing conditions (radioactivity, source-to-detector distance, and acquisition time) are listed in the table. It is worth noting that the simulation results of the measured counts of incident gamma-rays in Table 1 are obtained in an ideal environment without a natural background, while the experimental results are given after subtracting the natural background. As described in Section 2.1, the counts of effective 2-events in Table 1 can be obtained after the backscattering is eliminated. As such, the proportion of effective imaging events accounts for about 2% for the sources with different energies. The difference between the simulation and experimental results mainly comes from the scattering in the environment. Compared with selecting imaging events by setting the energy window in a typical Compton camera with two-layer structure [29], the proportion of effective imaging events in this prototype is increased by an order of magnitude because all the effective 2-events including fully absorbed and partially deposited energies are used.

**Figure 8:**
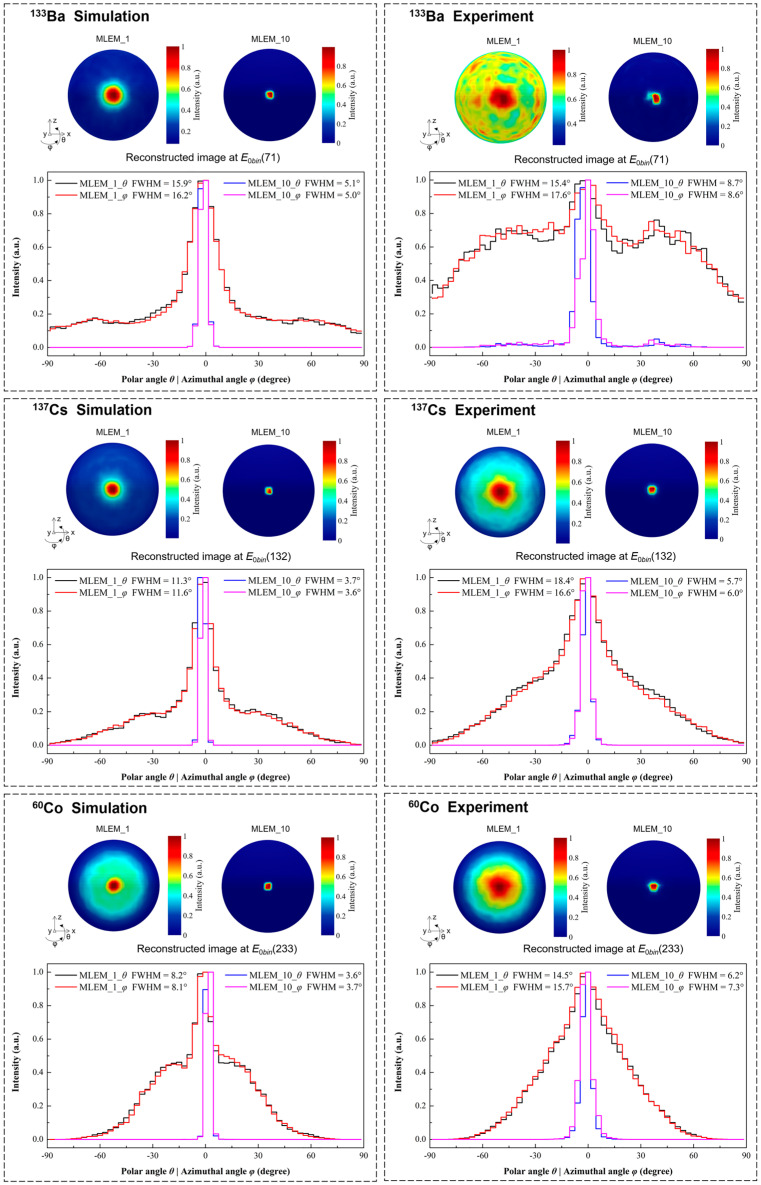
The reconstructed images of single point-like source (^133^Ba, ^137^Cs, ^60^Co) at the center of FOV with the conditions of simulations and experiments in Table 1. The iteration numbers of 1 and 10 are used to reconstruct the image. The profile plots taken through the maximum value of the reconstructed image on polar angle direction and azimuthal angle direction are shown.

The reconstructed images obtained under the test conditions in Table 1 are shown in Figure 8. The images with 1 and 10 iterations of MLEM are displayed. The image resolution is evaluated by the FWHM value, which is calculated from the profile plots taken through the maximum value on polar angle direction and azimuthal angle direction. The Gaussian fitting is used to obtain the FWHM value of the reconstructed hotspot. The simulated imaging resolutions of the ^133^Ba, ^137^Cs, and ^60^Co sources after 10 iterations are 5.1°, 3.7°, and 3.7° FWHM, respectively. And the experimental imaging resolutions of the ^133^Ba, ^137^Cs, and ^60^Co sources after 10 iterations are 8.7°, 6.0°, and 7.3° FWHM, respectively.

#### 3.1.2. Field of View

The ideal FOV of the prototype is 2π; that is, the polar angle *θ* ranges from −90° to 90° and the azimuthal angle *φ* ranges from −90° to 90°. However, the actual FOV of the system is mainly affected by the back projection reconstruction and cannot achieve the ideal situation. As can be seen from Section 2.1, each event in the system matrix will be back-projected to the imaging space to form a Compton ring. If the original image is a point-like source, then a blurred hotspot should be reconstructed by the accumulation of the Compton rings [30]. When a point-like source is positioned at the boundary of the FOV, only the Compton rings inside the FOV will overlap to form a hotspot. In this occasion, the other side of these Compton rings will also produce hotspot artifacts. When there is a natural background, poor event statistics, and scattering from the surrounding environment, this influence will cause the FOV to narrow. On the other hand, in image reconstruction, the deposited energy at the scatterer will be limited as *E*_s_ < *E*/(1 + 2*E*/*m*_e_*c*^2^), where *E* is the initial energy of the incident gamma-ray. The energy limitation of scatterer is manifested in the size of the Compton ring formed for each event. Obviously, the higher the initial energy, the smaller the radius of the ring. Therefore, the FOV of the high-incident-energy gamma-ray source is smaller than that of the low-incident-energy source.

**Figure 9:**
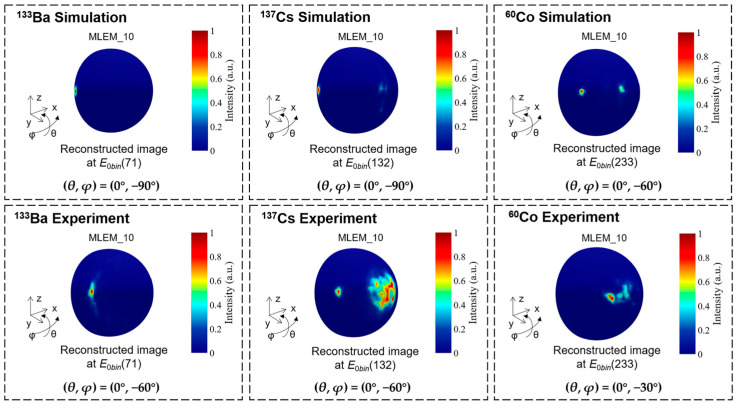
The simulation and experimental results of the reconstructed images after 10 iterations for a source (^133^Ba, ^137^Cs, ^60^Co) positioned at the boundary of field of view.

Figure 9 shows the simulation and experimental results of the reconstructed images for the ^133^Ba, ^137^Cs, and ^60^Co sources positioned at the boundary of the FOV after 10 iterations. The simulation results shows that the prototype can locate a point-like source of ^133^Ba, ^137^Cs, and ^60^Co at (*θ*, *φ*) = (0°, −90°), (0°, −90°), and (0°, −60°), respectively. This means that the ideal imaging FOV of the prototype can reach 180°, 180°, and 120° for ^133^Ba, ^137^Cs, and ^60^Co, respectively. However, due to the reasons mentioned above, the FOV in experimental tests cannot reach the optimal value. Another noteworthy reason is the “Jiyang Bagel” phenomenon [30]. If a hotspot can be reconstructed at the fully absorbed energy, it will become a bagel instead of a hotspot as the energy becomes lower. Therefore, when there is a high-energy background in the environment (such as ^40^K), it will inevitably affect the imaging FOV of the above radionuclides. The experimental results indicate the actual FOV of the prototype is 120°, 120°, and 60° for the ^133^Ba, ^137^Cs, and ^60^Co point-like sources, respectively, as shown in Figure 9.

#### 3.1.3. Imaging Sensitivity

**Figure 10:**
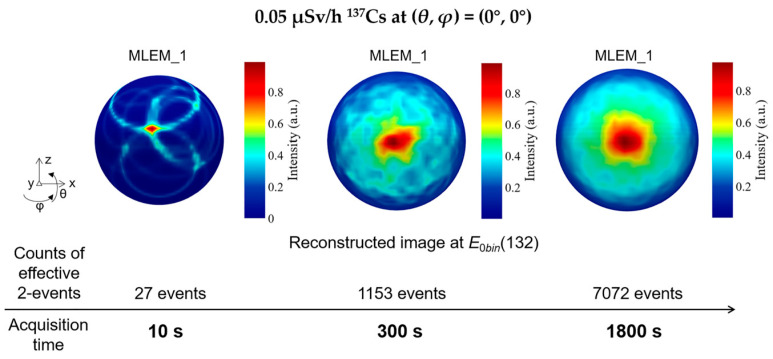
The reconstructed images with 1 iteration of MLEM for a 0.05 μSv/h ^137^Cs source at (*θ*, *φ*) = (0°, 0°). The hotspot can be preliminarily identified within 10 s.

The imaging sensitivity indicator is defined as the minimum exposure time necessary to correctly locate a single point source. In the experimental measurement, we placed a ^137^Cs point-like source with an activity of 1.88 MBq at a distance of 1.6 m in front of the detector. In this occasion, the produced dose rate from source at the detector position is about 0.05 μSv/h. The reconstruction process using the effective 2-events is shown in Figure 10. The number of MLEM iterations is 1 here. The hotspot is preliminarily identified within 10 s when 27 events are recorded as the effective 2-events. With the increase in acquisition time, the confidence of the reconstructed hotspot is gradually improved. When 1153 effective 2-events are collected within 300 s, the maximum value position of reconstructed hotspot is stable at (*θ*, *φ*) = (0°, 0°).

### 3.2. Multiple Point-like Source Imaging

This section presents the imaging tests performed with multiple point-like sources in the simulation and experiments. The radioactive sources are the same as those mentioned above.

#### 3.2.1. Angular Resolution

In a Compton camera, the angular resolution measure (ARM) is the distribution of the minimum angular distance between the known source position and Compton cones. In the experimental evaluation, the angular resolution can be estimated as the minimum angle for identifying two separate sources.

**Figure 11:**
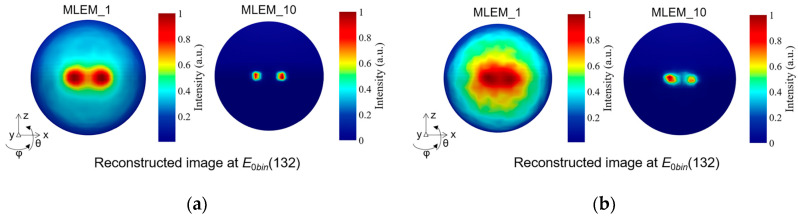
The reconstructed image of two ^137^Cs point-like sources separated by 15° for the measurement of angular resolution. (**a**) The simulation results. (**b**) The experimental results.

Figure 11 shows the measurement results of angular resolution in the simulations and experiments. Two ^137^Cs point-like sources are positioned at (*θ*, *φ*) = (0°, −7.5°) and (*θ*, *φ*) = (0°, 7.5°). The number of iterations of MLEM is set to 1 and 10. Two of the sources with an angular distance of 15° are roughly separated in the reconstructed image when using 1 iteration, whereas they are completely discriminated when using 10 iterations in both simulation and experimental measurement.

#### 3.2.2. Simultaneous Imaging of Three Point-like Sources

**Figure 12:**
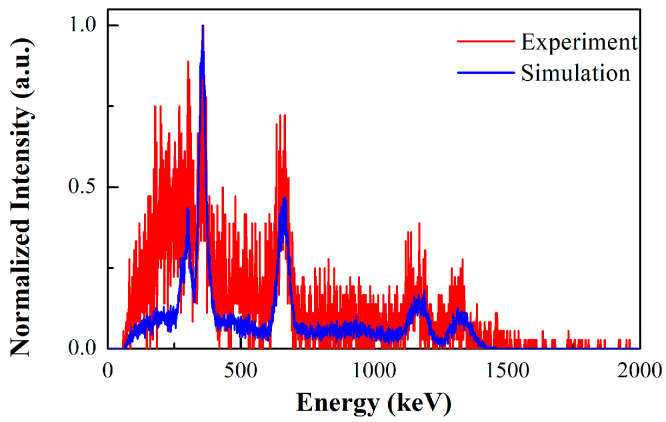
The coincidence summed energy spectra of the 2-events for the simultaneous measurement of 3 point-like sources (^133^Ba, ^137^Cs, and ^60^Co). Both the simulation and experimental results are shown.

The TLCC prototype can provide the image for each energy slice as well as the spectra for each direction. Thus, the capability of simultaneous separating radionuclides with various energies is evaluated. In the simulation and experimental measurements, we placed three point-like sources, ^133^Ba, ^137^Cs, and ^60^Co, at the emission directions of (*θ*, *φ*) = (0°, −45°), (0°, −10°), and (0°, 15°), respectively. The coincidence summed energy spectra of the 2-events for the simultaneous measurement of the three sources are shown in Figure 12. The experimental results of the distribution and relative intensity of the photopeaks of the three radionuclides are in good agreement with the simulation results.

**Figure 13:**
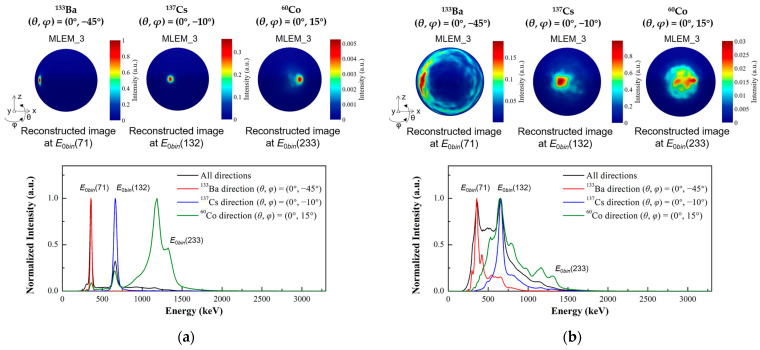
Multiple source localization with the prototype under the following test conditions: a ^133^Ba source positioned at (*θ*, *φ*) = (0°, −45°), a ^137^Cs source positioned at (*θ*, *φ*) = (0°, −10°), and a ^60^Co source positioned at (*θ*, *φ*) = (0°, 15°). The reconstructed images at the corresponding energy slices and the reconstructed initial energy spectra after 3 iterations are shown. The vertical axis of the reconstructed energy spectra is represented by normalized intensity. (**a**) The simulation results. (**b**) The experimental results.

The reconstructed images at the corresponding energy slices and the reconstructed initial energy spectra after three iterations are shown in Figure 13. The energy slices of *E*_0*bin*_(71), *E*_0*bin*_(132), and *E*_0*bin*_(233) represent the initial energies around 356 keV, 661.7 keV, and 1332.5 keV, respectively. The results demonstrate that the prototype correctly distinguishes the incident direction of each radionuclide. From the normalized intensity distribution of the reconstructed initial energy spectra, the photopeaks corresponding to the three nuclides can be distinguished. However, in the experimental results, the reconstruction of high-energy nuclides (^60^Co) is significantly influenced by low-energy nuclides (^137^Cs), which leads to the low photopeak intensity of high-energy nuclides and the relatively poor quality of the reconstructed image. Nevertheless, the incident direction of the high-energy nuclide can be correctly indicated when the corresponding energy slice is selected.

### 3.3. High-Energy Gamma-ray Imaging Evaluation

**Figure 14:**
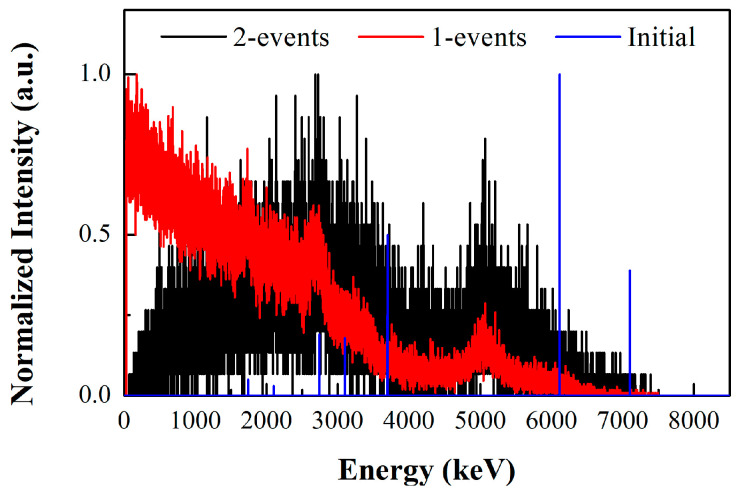
The measured single interaction (1-events) spectra and the coincidence summed energy spectra of the 2-events, in which the data are generated from a water target under 14.1 MeV neutron bombardment in the Geant4 simulation. The relative intensity of initial gamma-ray energies is indicated by the blue line.

In order to further evaluate the imaging ability of the prototype for higher-energy gamma-rays, we use the relative yield data of gamma-rays from the water target under 14.1 MeV neutron bombardment at 90° to the incident neutron beam [31] as original sources in the simulation, as shown by the blue line in Figure 14. Among them, the 6.13 MeV and 7.12 MeV gamma-rays are generated from the excitation of levels in ^16^O, and the 3.1 MeV and 3.7 MeV gamma-rays are generated by the de-excitation of the first two levels in ^13^C following the ^16^O(n, α)^13^C reaction.

In the simulation, a gamma-ray emitter with the above initial energies is positioned at a distance of 1 m in front of the detector. The measured single interaction (1-events) spectra and the coincidence summed energy spectra of the 2-events are shown in Figure 14. As can be seen, it is challenging to fully deposit incident energy through either a single interaction point or two interaction points for the gamma-rays with incident energies higher than 3 MeV. As such, using the traditional energy window method to select imaging events makes it difficult to correctly reconstruct the incident direction of a high-energy gamma-ray source. However, the energy-domain imaging method can resolve this conundrum by calculating the likelihood of incident energy in the energy-domain corresponding to each coincidence interaction event.

**Figure 15:**
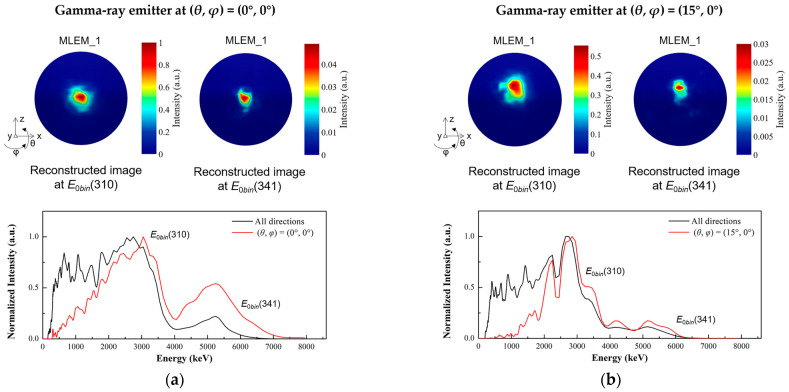
The reconstructed initial energy spectra and the reconstructed images with 1 iteration of MLEM for the water target under 14.1 MeV neutron bombardment in the Geant4 simulation. The energy slices of *E*_0*bin*_(310) and *E*_0*bin*_(341) represent the initial energy around 3100 keV and 6130 keV, respectively. (**a**) The gamma-ray emitter of water target is positioned at (*θ*, *φ*) = (0°, 0°). (**b**) The gamma-ray emitter of water target is positioned at (*θ*, *φ*) = (15°, 0°).

We placed a gamma-ray emitter at two different incident directions of (*θ*, *φ*) = (0°, 0°) and (15°, 0°) to evaluate the imaging capability of the prototype. Figure 15 shows the reconstructed initial energy spectra and the reconstructed images with one iteration of MLEM in the Geant4 simulation. Because of the high scattering platform, the photopeaks corresponding to the incident energies higher than 3 MeV are also difficult to display in the reconstructed initial energy spectra. Nevertheless, the images can be reconstructed at the corresponding energy slices. The energy slices of *E*_0*bin*_(310) and *E*_0*bin*_(341) represent the initial energy around 3.1 MeV and 6.13 MeV, respectively. Although 6.13 MeV energy has a low relative intensity in the reconstructed initial energy spectra, the reconstructed direction is consistent with 3.1 MeV. For both the incident direction of (*θ*, *φ*) = (0°, 0°) and (15°, 0°), the reconstructed images of the 3.1 MeV and 6.13 MeV energy slices all show the correct locating results. The simulation results demonstrate that the prototype can correctly indicate the 6.13 MeV source with various incident directions.

## 4. Discussion

The objective of this work is to develop a prototype of a three-layer Compton camera (TLCC), which consists of three GAGG-SiPM position-sensitive detectors. It is dedicated to applications in gamma-ray imaging with a wide energy range of 0.3~7 MeV. All detectors can distinguish the interaction positions in the 15 × 15 scintillator array. The averaged energy resolutions of the pixels are measured as (7.47 ± 0.58)%, (7.43 ± 0.61)%, and (8.37 ± 0.87)% at 661.7 keV for the three detectors, respectively.

The far-field energy-domain imaging method based on two interaction events is adopted, in which the space and spectral reconstruction are carried out. This method realizes the simultaneous estimation of the initial energy and spatial distribution of gamma-ray sources.

The simulation model of detectors is established based on the Geant4 Monte Carlo simulation toolkit. To optimize the effective detection efficiency and take angular resolution into account, the distance between three-layer detectors is designed for a wide range of gamma-ray energies. The imaging tests using ^133^Ba, ^37^Cs, and ^60^Co radionuclides under the conditions of single-point-like sources and multiple point-like sources are carried out with our prototype in laboratory measurements and compared with simulation studies.

The proportion of effective imaging events of TLCC accounts for about 2%, which is much higher than that using the traditional energy window method to choose imaging events. A 0.05 μSv/h ^137^Cs source at the center of the FOV is correctly identified in 10 s with our prototype. The imaging resolutions of the ^133^Ba, ^137^Cs, and ^60^Co sources at the center of the FOV after 10 iterations are 8.7°, 6.0°, and 7.3° FWHM, respectively. The angular resolution for resolving two ^137^Cs point-like sources is measured as 15°. It was also determined in the simulation that TLCC can obtain the reconstructed images of a 6.13 MeV source from different directions, in which the emitted gamma-ray data come from 14.1 MeV neutron scattering with water.

The simulation and experimental results show that the TLCC prototype has good imaging performance in a wide energy range. Future work will further test the imaging ability of the prototype under the experimental conditions of a higher-energy gamma-ray source.

## Figures and Tables

**Table 1:** The detection efficiency of TLCC for a point-like source at the center of FOV under the conditions of the simulation and experiment.

Settings of Gamma Emission Conditions		Nuclide	Representative Energy (keV)	Measured Counts of Incident Gamma-rays	Counts of Effective 2-Events	Proportion of Effective Events (%)
Simulation (*N* gammas emit within Ω = 0.031 sr at *d* = 1 m, *N* is equal to the sum of the emission probability of each energy branch multiplied by the number 10^7^)	*N* = 13,418,000	^133^Ba	356	760,871	11,266	1.48
	*N* = 8,499,000	^137^Cs	661.7	317,619	10,029	3.16
	*N* = 19,983,260	^60^Co	1173.2/1332.5	585,709	13,415	2.29
Experiment (Point-like source with radioactivity *A*, distance *d*, and acquisition time *t*)	*A* = 0.262 MBq *d* = 0.25 m *t* = 3600 s	^133^Ba	356	376,788	8253	2.19
	*A* = 1.88 MBq *d* = 1 m *t* = 1800 s	^137^Cs	661.7	628,321	13,186	2.10
	*A* = 1.92 MBq *d* = 1 m *t* = 1800 s	^60^Co	1173.2/1332.5	1,129,416	20,176	1.79

## Data Availability

The data that support the findings of this study are available from the corresponding author upon reasonable request.
